# Evidence confirms an anthropic origin of Amazonian Dark Earths

**DOI:** 10.1038/s41467-022-31064-2

**Published:** 2022-06-17

**Authors:** Umberto Lombardo, Manuel Arroyo-Kalin, Morgan Schmidt, Hans Huisman, Helena P. Lima, Claide de Paula Moraes, Eduardo G. Neves, Charles R. Clement, João Aires da Fonseca, Fernando Ozorio de Almeida, Carlos Francisco Brazão Vieira Alho, Christopher Bronk Ramsey, George G. Brown, Marta S. Cavallini, Marcondes Lima da Costa, Luís Cunha, Lúcia Helena C. dos Anjos, William M. Denevan, Carlos Fausto, Caroline Fernandes Caromano, Ademir Fontana, Bruna Franchetto, Bruno Glaser, Michael J. Heckenberger, Susanna Hecht, Vinicius Honorato, Klaus A. Jarosch, André Braga Junqueira, Thiago Kater, Eduardo K. Tamanaha, Thomas W. Kuyper, Johannes Lehmann, Marco Madella, S. Yoshi Maezumi, Leandro Matthews Cascon, Francis E. Mayle, Doyle McKey, Bruno Moraes, Gaspar Morcote-Ríos, Carlos A. Palheta Barbosa, Marcos Pereira Magalhães, Gabriela Prestes-Carneiro, Francisco Pugliese, Fabiano N. Pupim, Marco F. Raczka, Anne Rapp Py-Daniel, Philip Riris, Bruna Cigaran da Rocha, Leonor Rodrigues, Stéphen Rostain, Rodrigo Santana Macedo, Myrtle P. Shock, Tobias Sprafke, Filippo Stampanoni Bassi, Raoni Valle, Pablo Vidal-Torrado, Ximena S. Villagrán, Jennifer Watling, Sadie L. Weber, Wenceslau Geraldes Teixeira

**Affiliations:** 1 Institut de Ciència i Tecnologia Ambientals, Universitat Autònoma de Barcelona (ICTA-UAB), Bellaterra, Barcelona, Spain; 2 Geographical Institute, University of Bern, Bern, Switzerland; 3 Institute of Archaeology, University College London, London, UK; 4 Earth, Atmospheric and Planetary Sciences, Massachusetts Institute of Technology, Cambridge, MA USA; 5 Groningen Institute of Archaeology, University of Groningen, Groningen, Netherlands; 6 Museu Paraense Emílio Goeldi, Belém, Brazil; 7 Instituto de Ciências da Sociedade, Universidade Federal do Oeste do Pará, Santarém, Brazil; 8 Museum of Archaeology and Ethnology, University of São Paulo, São Paulo, Brazil; 9 Instituto Nacional de Pesquisas da Amazônia, Manaus, Brazil; 10 ArqueoMaquina, Belém, Brazil; 11 Departamento de Arqueologia, Universidade do Estado do Rio de Janeiro, Rio de Janeiro, Brazil; 12 Wageningen University & Research, Wageningen, Netherlands; 13 School of Archaeology, University of Oxford, Oxford, UK; 14 Embrapa Forestry, Colombo, Brazil; 15 Geosciences Institute, Federal University of Pará, Belem, Brazil; 16 Centro de Ecologia Funcional, Universidade de Coimbra, Coimbra, Portugal; 17 Soils Department, Federal Rural University of Rio de Janeiro, Seropédica, Brazil; 18 Department of Geography, University of Wisconsin-Madison, Gualala, CA USA; 19 Museu Nacional, Universidade Federal do Rio de Janeiro, São Cristóvão, Brazil; 20 Princeton Institute for International and Regional Studies, Princeton University, Princeton, NJ USA; 21 Naturalis Biodiversity Center, Leiden, Netherlands; 22 Embrapa Solos, Rio de Janeiro, Brazil; 23 Department of Soil Biogeochemistry, Martin-Luther-Universität Halle-Wittenberg, Halle, Germany; 24 Department of Anthropology, University of Florida, Gainesville, FL USA; 25 School of Public Affairs, UCLA, Los Angeles, CA USA; 26 Graduate Institute for International Development Research, Geneva, Switzerland; 27 Instituto de Desenvolvimento Sustentável Mamirauá, Tefé, Brazil; 28 School of Integrative Plant Science, Department of Global Development, Cornell University, Ithaca, NY USA; 29 Culture and Socio-Ecological Dynamics Research Group, Department of Humanities, Universitat Pompeu Fabra, Barcelona, Spain; 30 Institució Catalana de Recerca i Estudis Avançats (ICREA), Barcelona, Spain; 31 Department of Ecosystem and Landscape Dynamics, University of Amsterdam, Amsterdam, Netherlands; 32 Department of Archaeology, Max Planck Institute for the Science of Human History, Jena, Germany; 33 Faculty of Archaeology, Leiden University, Leiden, Netherlands; 34 Department of Geography and Environmental Science, University of Reading, Reading, UK; 35 CEFE, Univ Montpellier, CNRS, EPHE, IRD, Univ Paul-Valéry Montpellier, Montpellier, France; 36 Amazon Hopes Collective, Belém, Brazil; 37 Instituto de Ciencias Naturales, Universidad Nacional de Colombia, Bogotá, Colombia; 38 Institute of National Historic and Artistic Heritage, Belém, Brazil; 39 Departamento de Ciências Ambientais, Universidade Federal de São Paulo, Diadema, Brazil; 40 Institute for Modelling Socio-Environmental Transitions, Bournemouth University, Poole, UK; 41 Climate and Agriculture Group, Agroscope, Zurich, Switzerland; 42 French National Centre for Scientific Research, Paris, France; 43 Instituto Nacional do Semiárido (INSA), Campina Grande, Brazil; 44 Center of Competence for Soils, BFH-HAFL, Zollikofen, Switzerland; 45 Museu da Amazônia, Manaus, Brazil; 46 Soil Science Department, University of São Paulo, Piracicaba, Brazil

**Keywords:** Environmental sciences, Archaeology

**arising from** Silva et al. *Nature Communications* 10.1038/s41467-020-20184-2 (2021)

First described over 120 years ago in Brazil, Amazonian Dark Earths (ADEs) are expanses of dark soil that are exceptionally fertile and contain large quantities of archaeological artefacts. The elevated fertility of the dark and often deep A horizon of ADEs is widely regarded as an outcome of pre-Columbian human influence^1^. Archaeological research provides clear evidence that their widespread formation in lowland South America was concentrated in the Late Holocene, an outcome of sharp human population growth that peaked towards 1000 BP^2–4^. In their recent paper Silva et al.^5^ argue that the higher fertility of ADEs is principally a result of fluvial deposition and, as a corollary, that pre-Columbian peoples just made use of these locales, contributing little to their enhanced nutrient status.

Soil formation is inherently complex and often difficult to interpret, requiring a combination of geochemical data, stratigraphy, and dating. Although Silva et al. use this combination of methods to make their case^5^, their hypothesis, based on the analysis of a single ADE site and its immediate surroundings (Caldeirão, see maps in Silva et al.^5^), is too limited to distinguish among the multiple possible mechanisms for ADE formation. Moreover, it disregards or misreads a wealth of evidence produced by archaeologists, soil scientists, geographers and anthropologists, showing that ADEs are anthropic soils formed on land surfaces enriched by inputs associated with pre-Columbian sedentary settlement^6–9^. To be accepted, and be pertinent at a regional level, Silva et al.’s hypothesis^5^ would need to be supported by solid evidence (from numerous ADE sites), which we demonstrate is lacking.

## Geomorphological and pedological considerations

**Fig. 1:**
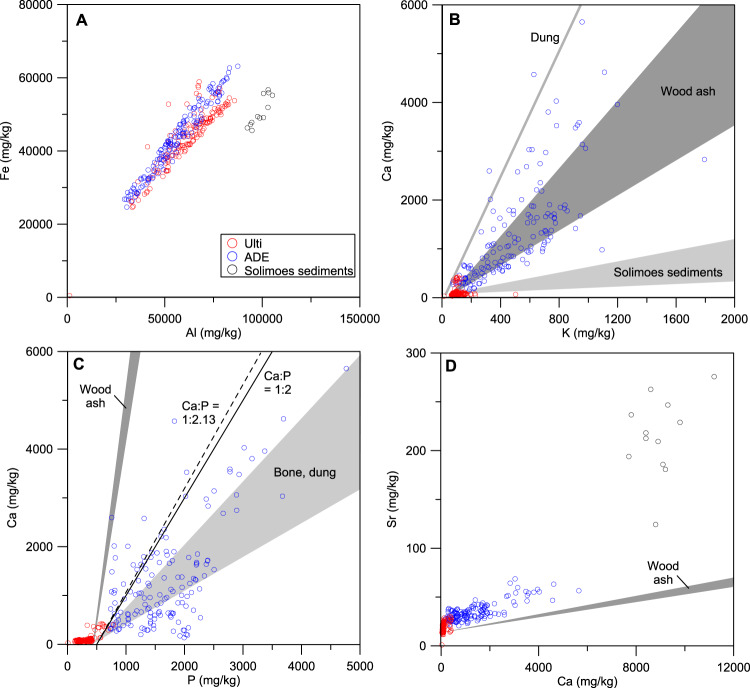
Caldeirão’s soil compositional data compared with published data of Solimões River sediments and anthropic materials. Data is in Supplementary Table 1; the wood ash and bone/dung fields in (**C**, **D**) are offset to compensate for soil (Ulti) background concentrations. ADE = Amazonian Dark Earth, Ulti = Ultisol soil profile. **A** Geogenic elements Al and Fe are similar in ADE and Ultisols, but different from Solimoes sediments. **B**, **C** Anthropogenic elements K, Ca, and P fall in the range of anthropogenic materials. Solimões sediments have much lower Ca/K ratios and far higher K concentrations. Black continuous and broken lines give the 1:2 and 1:2.13 Ca:P ratios quoted by Silva et al.^5^ for human faeces and freshwater fish, respectively, corrected for 500 mg/kg soil (Ulti) background. **D** Ca and Sr show strong correlations in ADE. The Ca/Sr ratio in ADE is close to that of wood ash, suggesting an anthropogenic origin for Sr, while Solimões sediments have overall much higher values.

There are several problems with reviving the argument^10^ that ADE fertility originates from deposited alluvium. First, the Caldeirão ADE site is located on a Miocene plateau ~20 m above the Solimões River floodplain (~40 m asl), which in itself precludes significant flooding during the Holocene^11^. Second, the parent material of the ADE and adjacent Ultisol shows analogous clay mineralogy and geogenic composition: both sites are characterised by the same 1:1 clays (as shown by Silva et al.’s Supplementary Fig. 3^5^) and both lack the 2:1 clay minerals expected from fluvial origin^12^. Moreover, no difference is observed in the geogenic elements (Al, Ti, Cr, V, Fe, As) (Fig. 1A). Third, the overall mineral assemblage of the Caldeirão ADE is incompatible with the geochemistry of the sedimentary load of the Solimões River (Fig. 1A, B, D). Fourth, the lower clay content in the anthropic ADE horizons at Caldeirão (erroneously described by Silva et al. as “sandy clay loam”^5^) is not evidence of fluvial deposition but a partial outcome of argilluviation^9^. Fifth, other well-studied ADE sites nearby contradict Silva et al.’s inference^5^: at the Hatahara ADE site, located 4 km from Caldeirão on the same Miocene bluff, the similarity in quartz sand grain morphology between the ADE A and B horizons excludes the inference of fluvial inputs into the A horizon^13^. Further afield, a large number of ADE sites are found along blackwater (non alluvial) rivers, associated with small headwater streams and springs, or found at elevations exceeding 90 m above the maximum flood level^14–16^, demonstrating that alluvial deposition is irrelevant to the formation of many ADE expanses. Indeed, if ADE were the result of alluvial processes, their spatial distribution along rivers would be continuous rather than patchy.

## Archaeological considerations

**Fig. 2:**
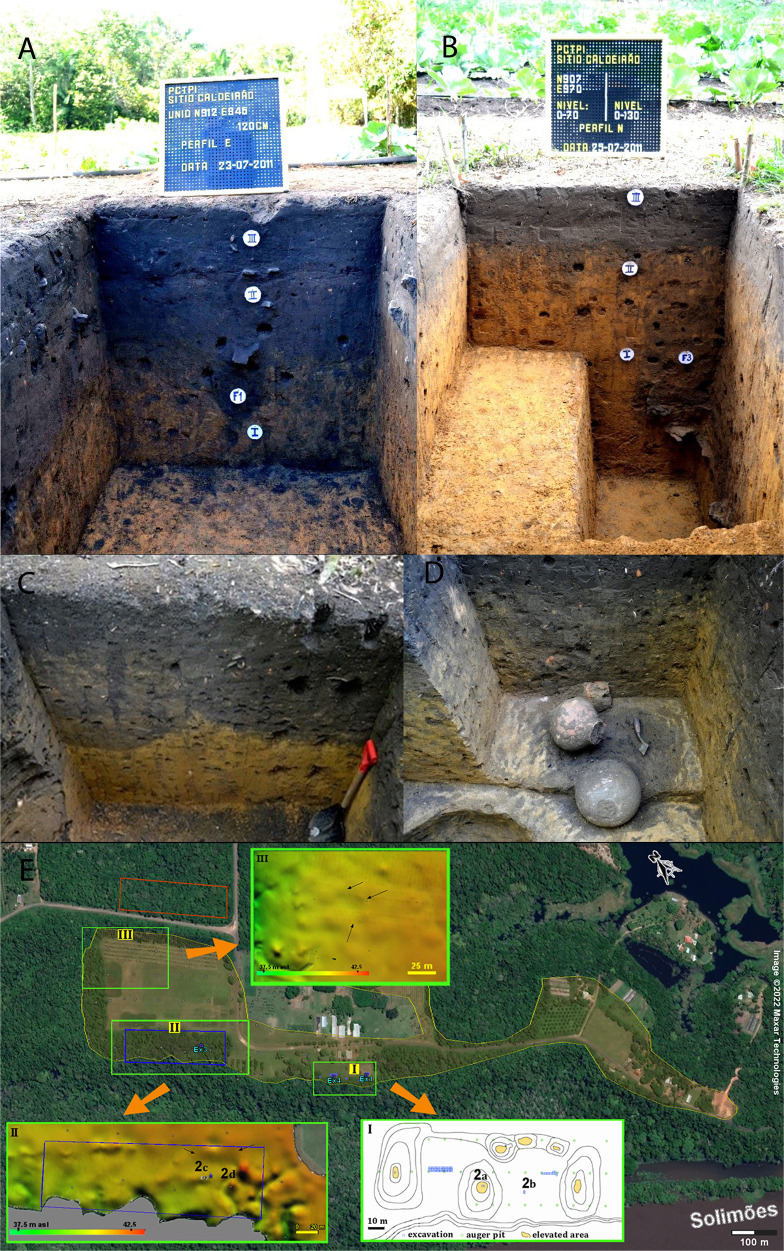
Archaeological fieldwork—excavations and mapping—carried out at the Caldeirão site in 2011. **A**, **B**, **C**, and **D** Vertical profiles exposed by multiple archaeological excavations at the Caldeirão ADE. **E** Google Earth image of the Caldeirão ADE (see location of profiles **A**–**D** within insets I and II). 2a and 2b are ~25 m apart and show the stratigraphy of archaeological deposits in mound (2a) and flat (2b) areas. 2c and 2d are ~12 m apart and show the stratigraphy of archaeological deposits at an Embrapa reference profile (**C**) and nearby archaeological excavation (**D**). Note clearly defined archaeological matrix features infilled with ADE sediment (**C**), and infilled pit feature with well-preserved ceramic vessels, suggesting intentional deposition by ancient indigenous Amazonians (**D**). **E** Yellow shaded area shows the spatial distribution of mounds and archaeological pottery ascertained through archaeological survey and excavation. Insets I, II, II show details of the topography and/or archaeological excavations, as well as sampling location for profiles depicted in (**A**–**D**). Inset II: Note the close proximity between identified mounded areas (black arrows), archaeological excavations, and the area of the ADE sampled by Silva^5^ (blue rectangle). Inset III: Survey has also identified mounded areas (black arrows) near the area Silva et al.^5^ sampled for Ultisols (red rectangle).

Research conducted at numerous archaeological sites in the Central Amazon^17^ has shown that the largest ADE expanses record multi-component occupations that date to the period 1200–800 BP and are often underlain by remains of older (<2500 BP) ceramic occupations^2–4,6^. This also applies in the case of the Caldeirão site, where coring and excavations clearly show that the ADE is a pottery-rich archaeological deposit characterised by a predominantly human-made assemblage of mounds and pits (Fig. 2A–E). Silva et al.’s sampling transects and elemental/isotopic measurements neither take into consideration nor detect this demonstrable anthropic conditioning of pre-Columbian origin (see Inset II in Fig. 2E)^5^. Furthermore, Silva et al. misunderstand stratigraphic associations when suggesting that >7.6 ky ^14^C BP charcoal collected from −90 cm in their Ultisol transect provides an accurate age marker for the beginning of ADE formation^5^. Middle Holocene charcoal fragments are commonly found stratified in Amazonian soil profiles^18^, including the B horizons of ADE profiles^14^. However, the relevant age to understand ADE formation (and whether it is consistent with human occupation) is that of the silt-sized charcoal making up the dark horizon of an ADE. At the nearby ADE site of Hatahara, the age of this charcoal pool is consistent with a late first millennium AD Paredão phase settlement, albeit with older occupations starting around 500 BC^19,20^. For Caldeirão, similar ages have been reported^21^.

## Demographic considerations

Silva et al. argue that a late Holocene onset for incipient agriculture in the Central Amazon region would preclude populations large enough to produce the levels of elemental enrichment recorded at Caldeirão^5^. This argument presupposes that indigenous land use regimes relying on incipient agriculture, aquatic wildlife, and hunting could not have created areas of persistent high fertility. This assumption does not account for decades of research on the subject. For instance, ethnoarchaeological research with the Kuikuro community, who are fisher-cultivators that live in the Upper Xingu region, has demonstrated that the greatest enrichment in P, Ca, and Sr, as well as high organic carbon and nearly neutral pH, occurs in mounded refuse middens. Once enriched soil horizons form in the middens, typically within a few years, they are often used for cultivating crops such as maize, sweet potato, and manioc^22^. Soil enrichment and ADE formation, therefore, are consistently associated with domestic activities in indigenous villages and, contrary to Silva et al.’s claim^5^, it is this elemental enrichment accumulating in settlements that is used for cultivation (and not the other way around). More broadly, measurements of elemental enrichment with P and Ca constitute a poor demographic proxy and, on their own, do not reveal agricultural activity: virtually any long human occupation can result in soil enrichment^23^. ADE sites, like Caldeirão, are very rich in nutrients because they concentrate human debris and waste associated with resources gathered or produced in large areas. It is the concentration of resources in settlements that produce ADEs over hundreds or thousands of years. Put another way, a thousand people could extract resources produced from a 50 hectares’ catchment but concentrate debris and waste in a village of 0.1 hectares. Silva et al.’s^5^ reference to improbably large agricultural populations, which implicitly suggests that ADEs were initially established for agricultural purposes, does not constitute evidence of fluvial deposition and disregards the association between ADE and middens that is supported by current research.

## Elemental enrichment and isotopic ratios of ADE vs. Ultisols (Acrisols)

Most of the co-authors of Silva et al.^5^ have elsewhere argued that the elemental composition of Caldeirão site “…can be used to unveil ADE sites and differentiate them from Amazonian soils without anthropic influence”^24^. We agree with their earlier assessment: enrichment of the ADE compared to the Ultisols is consistent with inputs associated with human settlement. Among the latter are those related to burning, including K, Rb, Ba, Ca, Sr, P (from ash and charcoal); P, Ca, Sr, K, Zn, Cu (human waste); and Ca, P, Sr, Zn (bone debris) (Fig. 1B, C)^25^. Most of these, along with pyrogenic C, have been reported in ADEs^8^. The most logical explanation for such an assemblage is anthropic inputs associated with settlement activity. Indeed, research at the Hatahara site shows that the dark ADE sediments are bulked up by sand and silt-sized particulate material resulting from anthropic activity (fragmented charcoal and bone, pottery fragments, sponge spicules, etc.)^13^. Bioturbation can then mix these added materials in soil over time throughout the profile. How, then, can a fluvial input be surmised? The core of Silva et al.’s argument is that differences in Sr and Nd isotope ratios between ADE and Ultisols are best explained by fluvial inputs^5^. However, both Sr and Nd are found in plants^26^ and terrestrial and aquatic vertebrates^27^, as well as in mineral matter and Silva et al. admit that their methods cannot discriminate these sources^5^. As there are no independent indications of sediment input in ADE’s bulk chemical composition, but ample evidence for non-mineral anthropogenic inputs, it is most likely that isotopic signature in the studied ADE resulted from the deposition of food debris. Silva et al. regard the difference in elemental stoichiometries of freshwater fish (Ca:P ~2.13) and human faeces (Ca:P ~2) compared with ADEs as further evidence of ADE being of fluvial origin^5^. However, while the Ca:P ratio is highly variable in Caldeirão ADE (Fig. 1C), the modern Ca:P ratio in ADEs is the result of differential preservation coupled with the specific tropical soil dynamics of Ca, which is easily leached, and P, which binds with soil Fe and Al oxides^28^.

By way of conclusion: the geogenic model for ADE formation, which famously argued that ADEs are dark soils of natural fertility resulting from the deposition of alluvial horizons^10^, was laid to rest over 40 years ago^29^. Silva et al.’s hypothesis^5^ reiterates this geogenic position but, as we have shown here, it does not stand up to scrutiny.

## Supplementary information

Supplementary Information

## Data Availability

All relevant data are provided with the paper.
