# Supplementary material for: Evidence confirms an anthropic origin of Amazonian Dark Earths
Source: Nat Commun. 2022 Jun 17;13:3444. doi: 10.1038/s41467-022-31064-2 (PMC9205880; doi:10.1038/s41467-022-31064-2)
Supplement: Supplementary file 1 — Supplementary Information [file 41467_2022_31064_MOESM1_ESM.pdf]

Evidence confirms an anthropic origin of Amazonian Dark Earths

Lombardo et al.

Supplementary table 1

Table 1 Data used in Figure 1 based on Silva et al.[1], Viers et al.[2], Braadbaart et al.[3], Cílová and Woitsch[4], Vassilev et al.[5], Huisman et al.[6]

Material	K mg/kg	Ca mg/kg	P mg/kg	Al mg/kg	Fe mg/kg	Sr mg/kg	Source*
Beech ash	159480	281629	14408	4765	4895	1099	Cilova & Woitsch 2012 (table 2)
Beech ash	130883	330950	14408	14294	15383	1522	Cilova & Woitsch 2012 (table 2)
Spruce ash	63792	360971	10479	13235	11188	1691	Cilova & Woitsch 2012 (table 2)
Spruce ash	52793	390993	11789	19059	11188	1607	Cilova & Woitsch 2012 (table 2)
Wood ash s.l. (average)	59117	307576	15194	26947	24054	NA	Vassilev et al. 2013 (table 6)
Wood ash s.l.	33137	230164	26000	6000	4995	NA	Braadbaart et al. 2012 (table 3)
Ash of cow dung	7290	48034	45000	8000	4995	NA	Braadbaart et al. 2012 (table 3)
Unaltered bone (Stavanger, N)	0	145747	180366	16300	77000	NA	Huisman et al. (2017) (table 3)
Unaltered bone (Stavanger, N)	0	138384	178359	12900	81500	NA	Huisman et al. (2017) (table 3)
Unaltered bone (Stavanger, N)	0	103788	152401	16200	110600	NA	Huisman et al. (2017) (table 3)
Unaltered bone (Zug, CH)	0	282630	235493	0	33900	NA	Huisman et al. (2017) (table 3)
Unaltered bone (Zug, CH)	0	252108	206056	0	33900	NA	Huisman et al. (2017) (table 3)
Unaltered bone (Zug, CH)	0	267977	211676	3500	30900	NA	Huisman et al. (2017) (table 3)
Suspended sediment Solimoes river	19800	8800	NA	98700	49000	124	Viers et al. (2008) (table 1a)
Suspended sediment Solimoes river	21100	7800	NA	102900	51900	237	Viers et al. (2008) (table 1a)
Suspended sediment Solimoes river	19900	9200	NA	94700	45600	181	Viers et al. (2008) (table 1a)
Suspended sediment Solimoes river	18200	8400	NA	94600	47800	218	Viers et al. (2008) (table 1a)
Suspended sediment Solimoes river	18800	7700	NA	93900	47200	194	Viers et al. (2008) (table 1a)
Suspended sediment Solimoes river	20200	11200	NA	105200	55200	276	Viers et al. (2008) (table 1a)
Suspended sediment Solimoes river	18600	9800	NA	103300	55900	229	Viers et al. (2008) (table 1a)
Suspended sediment Solimoes river	19300	9300	NA	100600	55700	247	Viers et al. (2008) (table 1a)
Suspended sediment Solimoes river	18400	8600	NA	103000	56700	263	Viers et al. (2008) (table 1a)
Suspended sediment Solimoes river	17000	8900	NA	92300	46300	209	Viers et al. (2008) (table 1a)
Suspended sediment Solimoes river	18600	8400	NA	100600	49100	213	Viers et al. (2008) (table 1a)
Suspended sediment Solimoes river	18300	9100	NA	97600	49400	186	Viers et al. (2008) (table 1a)

*Where necessary, data were recalculated from percent or oxide percent to mg/kg

1 Silva, L. C. R. *et al.* A new hypothesis for the origin of Amazonian Dark Earths. *Nat. Commun.* **12**, 127, doi:10.1038/s41467-020-20184-2 (2021).

2 Viers, J. *et al.* Seasonal and provenance controls on Nd–Sr isotopic compositions of Amazon rivers suspended sediments and implications for Nd and Sr fluxes exported to the Atlantic Ocean. *Earth and Planetary Science Letters* **274**, 511-523, doi:https://doi.org/10.1016/j.epsl.2008.08.011 (2008).

3 Braadbaart, F., Poole, I., Huisman, H. D. J. & van Os, B. Fuel, Fire and Heat: an experimental approach to highlight the potential of studying ash and char remains from archaeological contexts. *Journal of Archaeological Science* **39**, 836-847, doi:https://doi.org/10.1016/j.jas.2011.10.009 (2012).

4 Cílová, Z. & Woitsch, J. Potash – a key raw material of glass batch for Bohemian glasses from 14th–17th centuries? *Journal of Archaeological Science* **39**, 371-380, doi:https://doi.org/10.1016/j.jas.2011.09.023 (2012).

5 Vassilev, S. V., Baxter, D. & Vassileva, C. G. An overview of the behaviour of biomass during combustion: Part I. Phase-mineral transformations of organic and inorganic matter. *Fuel* **112**, 391-449, doi:https://doi.org/10.1016/j.fuel.2013.05.043 (2013).

6 Huisman, H., Ismail-Meyer, K., Sageidet, B. M. & Joosten, I. Micromorphological indicators for degradation processes in archaeological bone from temperate European wetland sites. *Journal of Archaeological Science* **85**, 13-29, doi:https://doi.org/10.1016/j.jas.2017.06.016 (2017).
